# Sulfonated Starch-*Graft*-Polyaniline@Graphene Electrically Conductive Nanocomposite: Application for Tyrosinase Immobilization

**DOI:** 10.3390/bios12110939

**Published:** 2022-10-28

**Authors:** Marzieh Aliya, Ehsan Nazarzadeh Zare, Hassan Faridnouri, Matineh Ghomi, Pooyan Makvandi

**Affiliations:** 1 School of Chemistry, Damghan University, Damghan 36716-41167, Iran; 2 School of Biology, Damghan University, Damghan 36716-41167, Iran; 3 Centre for Materials Interface, Istituto Italiano di Tecnologia, Viale Rinaldo Piaggio 34, Pontedera, 56025 Pisa, Italy

**Keywords:** sulfonated starch, polyaniline, graphene, electrical nanocomposite, tyrosinase

## Abstract

The interaction of tyrosinase with sulfonated starch-*graft*-polyaniline@graphene (SSt-*g*-PANI@G) nanocomposite was investigated by electrochemical methods. The activity of the immobilized tyrosinase (Tyase) was proved by the electrochemical detection of three substrates (L-dopa, caffeic acid, and catechol). The SSt-*g*-PANI@G nanocomposite was characterized by Fourier-transform infrared spectra (FT-IR), X-ray diffraction (XRD), field emission scanning electron microscopy (FESEM), energy-dispersive X-ray analysis (EDX), and thermogravimetric analysis (TGA). To immobilize tyrosinase on the surface of the nanocomposite, a simple drop-casting technique was used. The presence of sulfuric acid and hydroxyl groups in SSt, amine groups in PANI, and high surface-to-volume ratio and electrical conductivity of graphene in the prepared nanocomposite led to good enzyme immobilization on the electrode surface. The modified electrode showed a suitable catalytic effect on the electrochemical redox agent, compared with the bare electrode. The peak current responses for three substrates were studied with a calibration curve derived using cyclic voltammetry (CV) and differential pulse voltammetry (DPV). In addition, the fabricated SSt-g-PANI@G/Tyase/GCE showed a more suitable response to catechol, L-dopa, and caffeic acid substrates, respectively.

## 1. Introduction

Electrochemical technique studies of the redox behavior of different compounds in chemical reactions usually offer high sensitivity, good selectivity, rapid response, and low cost. Electrochemical biosensors are considered a promising method for phenolic compound detection, based on the immobilization of bioreceptors on the electrode surface [1,2]. In biosensor development, immobilization is a key, important step, which involves the application of a novel sensing material with good electronic properties that is biocompatible, stable, easily accessible by the analyte, and has a large surface area [3]. Enzyme stabilization techniques encompass van der Waals, ionic, and covalent interactions. This sort of biosensor can be developed utilizing various enzymes such as polyphenol oxidase, peroxidase, amine oxidase, and tyrosinase [4,5,6]. Many studies on electrochemical sensors and biosensors have been conducted over the last few decades due to the possibility of merging speed, selectivity, and sensitivity in low-cost chemical analysis techniques [7,8,9,10].

Tyrosinase (Tyase) is a blue copper protein (with two copper atoms in the active center), which can be considered a polyphenol oxidase. Tyrosinase biosensors, depending on their specificity, are used for the detection of phenolic compounds in the food industry. 

This important enzyme catalyzes two consecutive oxidation reactions: (1) the o-hydroxylation of phenols to guaiacol and, subsequently, (2) the oxidation of guaiacol to o-quinones, both in the presence of molecular oxygen. Furthermore, Tyase has been extensively used in biosensor construction for the determination of phenols [11,12].

On the other hand, the immobilization of biomolecules such as proteins or enzymes on electrical conductive polymer matrixes for biosensing is a promising direction given the rapid advances in biotechnology [13,14]. This approach provides a simple, low-cost method for protein immobilization and redox proteins show enhanced electrochemical activity, allowing electrochemical measurement of their substrate with higher sensitivity and better selectivity.

Electrically conductive polymers have piqued the interest of researchers in recent years due to their excellent electrical conductivity and chemical stability [15,16,17]. Among conductive polymers, polyaniline (PANI) is of tremendous interest due to its ease of fabrication, low cost, availability, excellent electrical conductivity, and good environmental stability. Moreover, PANI displays two redox couples which ease the charge transfer between an enzyme and a polymer. Due to the great electrochemical properties along with in vivo biocompatibility, PANI-based nanocomposites can be used to detect a negligible amount of biomolecules with high sensitivities and fast responses [18,19,20,21,22]. To enhance the good immobilization of enzymes on the PANI, the copolymerization method with functionalized monomers and/or modified natural polymers can be used. Although this method leads to decreasing the electrical conductivity of PANI, it can use the inherent electrically conductive materials such as graphene (G) in the PANI copolymer matrix.

Graphene (G) has ideal and unique properties such as excellent conductivity, large surface area, good chemical stability, mechanical strength, and high charge transport mobility. Therefore, it has attracted significant interest in several technological application fields including nanoelectronics, nanocomposite, biosensing, and bioelectronics [23,24,25].

This study developed a straightforward method for the fabrication of a sulfonated starch-*graft*-polyaniline@graphene (SSt-*g*-PANI@G) nanocomposite and immobilized Tyase on its surface. In addition, we presented a new electrochemical sensor for the measurement of three substrates (L-dopa, caffeic acid, and catechol). The SSt-*g*-PANI@G nanocomposite was fabricated by in situ copolymerization. The nanocomposite was dropped onto the clean and polished surface of the glassy carbon electrode surface, and then Tyase was immobilized on the SSt-*g*-PANI@G nanocomposite surface. The morphology, structure, and features of the prepared nanocomposite were investigated via XRD, FESEM, EDX, FT-IR, and TGA. The SSt-*g*-PANI@G/Tyase was employed for the detection of substrates such as L-dopa, caffeic acid, and catechol.

## 2. Material and Approaches

### 2.1. Materials

Corn starch was purchased from a local store. Graphene nanoparticles with a diameter of 20–30 nm (95% < purity) were purchased from Notrino, Iran. Chlorosulfonic acid, polyaniline, hydrochloric acid 37%, ammonium persulfate (as an initiator), chloroform, dimethyl sulfoxide (chromatographic purity), acetic acid, sodium acetate, acetone, and methanol were acquired from Merck, Germany. Tyrosinase (from mushroom, EC 1.14.18.1; activity 2700 U/ mg of solid) and L-dopa were also purchased from Sigma Co., Ltd. (St. Louis, MO, USA) and used without further purification. All solutions were prepared with double-distilled water.

### 2.2. Apparatus

An Equinox55 spectrometer was used for the FT-IR analysis (Bruker, Karlsruhe, Germany). TGA analyses were performed using the NETZSCH TG209F3 calorimeter (Germany) by scanning up to 800 °C. The X-ray diffraction (XRD, BrukerD8 Advance, Germany) patterns were obtained at room temperature using Cu-Kβ radiation with a sweep speed of 5° min^−1^, and the patterns were recorded in the 2θ range of 5–70°. A digital pH meter (780 pH meter, Metrohm) with a precision of ±0.001 was applied to adjust the pH value of all solutions. Field emission scanning electron microscopy (FE-SEM)/EDX was recorded on a MIRA 3-XMU. Elemental analysis was carried out using a Leco CHNS-932 under room temperature. Autolab electrochemistry equipment with potentiostat/galvanostat/impedance analysis (AUTOLAB PGSTAT-30, Eco-Chemie, Utrecht, Netherlands) was employed for electrochemical impedance spectroscopy (EIS). EIS was carried out in the presence of 5.0 mM K_3_[Fe(CN)_6_]/K_4_[Fe(CN)_6_] in 0.1 M KCl as a redox probe in the frequency range of 0.10 Hz–100 kHz, amplitude of 5.0 mV. Voltammetry measurements were performed using a potentiostat/galvanostat (Kianshar Danesh, RADstat 10, Iran) that was controlled by a computer. The reference electrode was a Ag/AgCl (3 M KCl), while the auxiliary electrode was a platinum wire. As the working electrode, a glassy carbon electrode, GCE (Azarelectrode, Iran), with a geometrical area of 0.0314 cm^2^ (bare or modified), was employed. Before conducting the electrochemical measurements, electrolyte solutions were deoxygenated by purging inert nitrogen gases for 15 min. All experiments were carried out in a working solution (a 0.1 M phosphate buffer of pH = 6.8) at 25 ± 2 °C.

### 2.3. Sulfonated Starch Preparation

**Figure 1:**
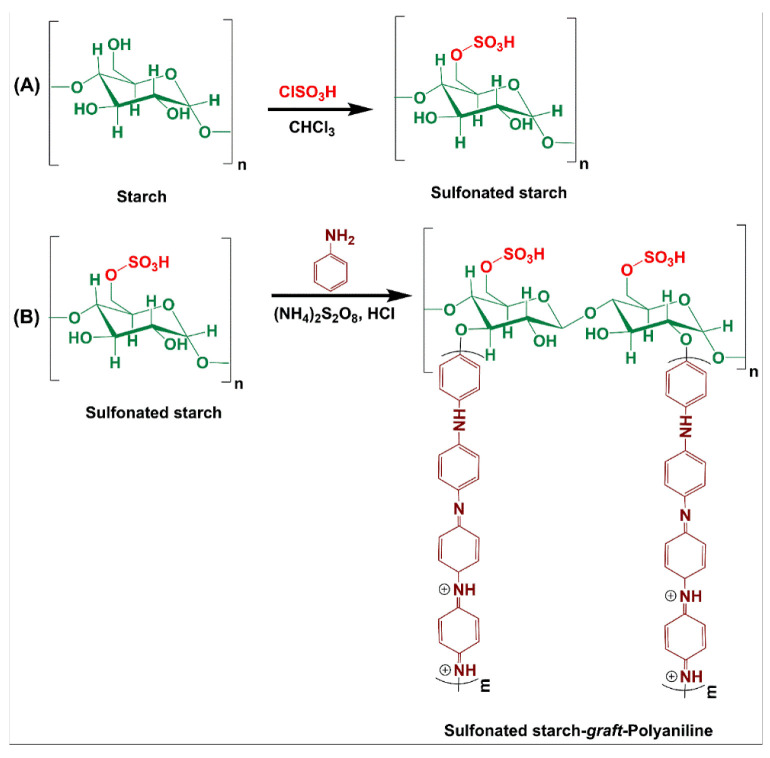
Preparation of sulfonated starch (**A**) and sulfonated starch-graft-polyaniline (**B**).

Sulfonated starch (SSt) was prepared as described in a previously reported lecture with some modifications [19]. First, 5.00 g of starch was added to 20 mL of chloroform (as a solvent), thoroughly mixed, and then homogenized by a magnetic stirrer. Next, 1.00 g of chlorosulfonic acid (equal to 0.8 mL) was dropwise added to a certain amount of chloroform solvent (2 mL) at 0 °C (in an ice bath) over 2 h. The mixture was placed on a magnetic stirrer for another 2 h at room temperature until the HCl gas was completely removed from the system and the functionalized centers increased. Then, the obtained mixture was centrifuged, filtered, and washed with water and methanol (30 mL, 1:1). Finally, the milky precipitate was dried at room temperature. The synthetic pathway reaction of SSt is shown in Figure 1A.

### 2.4. Preparation of Starch-Graft-Polyaniline (SSt-g-PANI)

SSt-g-PANI was synthesized via a thermally initiated free-radical polymerization. Briefly, 1.25 g of SSt was transferred into a 250 mL round-bottom flask along with 50 mL of distilled water. To complete the dissolution of starch, the mixture was stirred on a magnetic heater for 30 min at 50 °C. Then, it was cooled to room temperature and degassed in a nitrogen gas atmosphere for 15 min. In the next step, 3.75 mL hydrochloric acid (37%) was added to the flask under a constant temperature of 0–5 °C. To prepare the initiator solution, 3 g of ammonium persulfate (APS) initiator was dissolved in 20 mL of distilled water. Then, the initiator solution was added dropwise to the reaction vessel for 15 min. The reaction container was kept at a steady state for 10 min to generate active radical centers. Then, 2.5 mL aniline monomer was added to the reaction container and completely stirred at room temperature under nitrogen gas for 12 h to complete the copolymerization reaction. Finally, an obtained deep-green precipitate was filtrated and washed with methanol and distilled water several times. The product was dried in a vacuum oven at 50 °C (Figure 1B). To separate the homopolymer of polyaniline, the formed precipitate was transferred to 20 mL dimethyl sulfoxide solvent for 2 h. The remaining precipitate was washed with distilled water and acetone once and dried at 50 °C. The following equations were used to obtain the grafting percentage (G%) and efficiency percentage (E%) of SSt grafted to polyaniline:

(1) G% = (W_1_ − W_0_)/W_0_ × 100
(2) E% = (W_1_ − W_0_)/W_2_ × 100

where W_0_, W_1_, and W_2_ are the weight of SSt (g), the weight of graft copolymer after separation of homopolymer (g), and the total weight of the grafted copolymer and homopolymer (g), respectively. Based on these equations, the grafting percentage (G%) and efficiency percentage (E%) were 31% and 20%, respectively.

### 2.5. Fabrication of SSt-Graft-Polyaniline@Graphene (SSt-g-PANI@G) Nanocomposite

**Figure 2:**
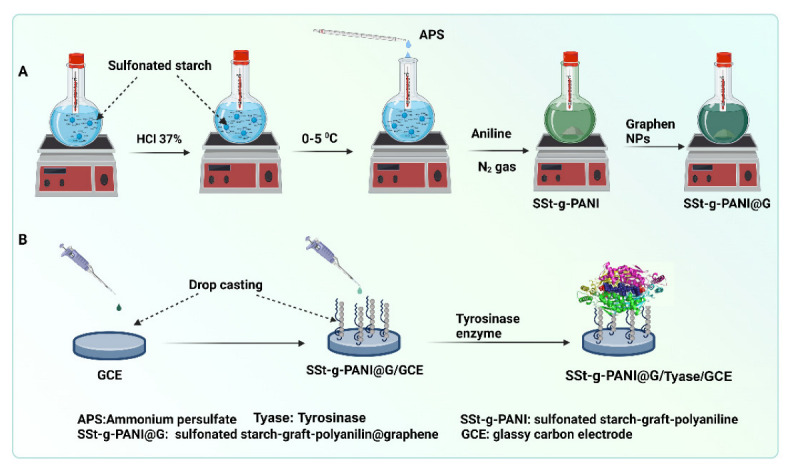
Schematic illustration of in situ copolymerization method for the fabrication of SSt-g-PANI@G nanocomposite (**A**) and electrode fabrication and enzyme immobilization method (**B**).

The nanocomposite was fabricated by the in situ copolymerization method as follows: an amount of the prepared SSt (1.75 g) was completely dissolved in 50 mL of distilled water. Afterward, the mixture was cooled at room temperature and degassed with nitrogen gas for 15 min. In another flask, 0.175 g of graphene (10 wt% to polymer) was sonicated in 15 mL of distilled water for 15 min. This reaction mixture was added to a container containing the SSt solution. The reaction mixture was exposed to nitrogen gas at 0–5 °C. Then, the initiator solution (2 g ammonium persulfate initiator in 50 mL distilled water) was added drop by drop to the reaction container for 15 min. In the next step, 2.5 mL aniline monomer was added to the reaction vessel and placed on a magnetic stirrer at room temperature under nitrogen gas for 12 h to complete the copolymerization reaction. Finally, the composite precipitate was separated by a centrifuge and washed several times with distilled water and methanol and then dried at 50 °C in a vacuum oven (Figure 2A).

### 2.6. Preparation of Modified Electrode and Enzyme Immobilization

To facilitate the stabilization of the nanocomposite on the glassy carbon electrode (GCE), the nanocomposite (1 mg) was sonicated in 1 mL DMSO solvent to form a homogeneous suspension. Afterward, 10 μL of the nanocomposite solution was dropped onto the clean and polished surface of the working electrode. To stabilize the nanocomposite on the electrode surface, the electrode was immersed in a vial containing the prepared nanocomposite solution for 30 min and was dried in the air. This process was repeated three times to reach a suitable thickness of the composite on the electrode surface. (The number of three times immersion was obtained experimentally in the laboratory, which obtained the best result). For enzyme immobilizing, 10 µL of Tyase (2.5 mg/mL) in PBS at pH 6.8 was dropped onto the surface of the GCE electrode modified with the nanocomposite. The electrode was placed in the refrigerator for 24 h for better adhesion and stabilization of the Tyase via the intermolecular interaction between the nanocomposite and the enzyme. The obtained electrode was labeled as SSt-*g*-PANI@G/Tyase/GCE (Figure 2B). 

The Tyase-immobilized electrode was stored at 4 °C when not in use.

## 3. Results

In the past few decades, conductive polymers have been exploited as excellent materials for preparing nanocomposites with special features (such as various functionality, appropriate stability, electrical conductivity, biocompatibility, etc.), in the design of various electrochemical biosensors. [20,21,22]. In this regard, we designed a biocompatible nanocomposite from sulfonated starch grafted to polyaniline and graphene for the immobilization of tyrosinase.

### 3.1. Characterization of Nanocomposite

**Figure 3:**
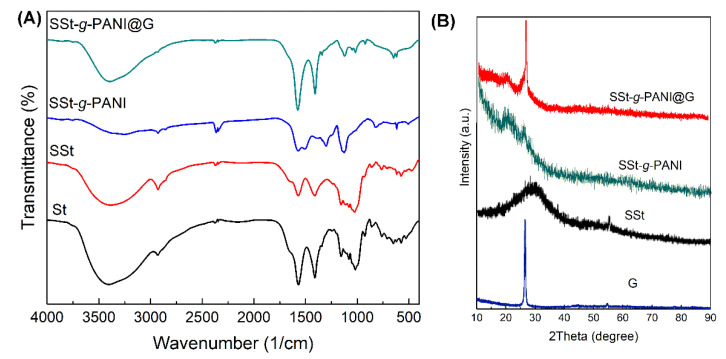
(**A**) FT-IR spectra of starch (St), sulfonated starch (SSt), sulfonated starch-*graft*-polyaniline copolymer (SSt-*g*-PANI), and sulfonated starch-graft-polyaniline @graphene (SSt-*g*-PANI@G) nanocomposite. (**B**) The XRD patterns of graphene (G), SSt, SSt-*g*-PANI, and SSt-*g*-PANI@G.

FT-IR spectroscopy was employed to characterize the SSt-*g*-PANI@G nanocomposite (Figure 3A). The FT-IR spectrum of a pure starch illustrates two absorption bands around 1081 cm^−1^ and 1156 cm^−1^ that are assigned to the C-O stretching vibration of C-O-H. As well, the band at around 1020 cm^−1^ was attributed to the C-O stretching vibrations of the C-O-C group [23]. The broad band at 6393 cm^−1^ can be assigned to the O-H stretching vibrations [24]. The absorption bands at 3392 cm^−1^ and 657 cm^−1^ refer to the OH stretching vibrations [25]. It can be seen from FT-IR of pure polyaniline (Figure S1) that the absorption bands around 1562 cm^−1^ and 1481 cm^−1^ were assigned to C=N and C=C stretching vibration in the benzonoid and quinonoid rings and the broadband at 3432 cm^−1^ was attributed to the N-H stretching vibrations [26,27]. By comparing the FT-IR spectra of pure starch and SSt, three new bands can be seen in the regions of 1276 cm^−1^, 2512 cm^−1^, and 590 cm^−1^, which are related to the asymmetric and symmetric O=S=O stretching vibrations and S-O stretching vibrations in sulfone functional groups, respectively [19]. In the FTIR spectra of SSt-*g*-PANI, the absorption bands at 1562 cm^−1^ and 1481 cm^−1^ related to polyaniline and the absorption bands around 1276 cm^−1^ and 2512 cm^−1^ related to SSt are observed with a slight shift and overlap of the peaks. On the other hand, the absorption bands at 3400 cm^− 1^, 1220 cm^−1^, and 1050 cm^−1^ correspond to OH, C=O, and C-O stretching due to carbonyl groups in the graphene [26]. In summary, the absorption bands related to SSt, polyaniline, and graphene are also well demonstrated, exhibiting a slight shift, confirming the successful formation of SSt-*g*-PANI@G nanocomposites.

Following the FT-IR analysis, the XRD pattern analysis was used to study the crystallinity and irregularity of graphene (G), sulfonated starch (SSt), sulfonated starch-graft- polyaniline (SSt-*g*-PANI), and SSt-*g*-PANI@G (Figure 3B). In the XRD pattern of sulfonated starch compared with the pure ones, the crystallinity lowered, which is related to the modification of the starch structure [23,24]. In the XRD pattern of pure polyaniline, comparatively wide peaks were also detected, which suggest the disordered structure of polyaniline [27,28]. Two distinct peaks were observed in the XRD pattern of graphene at 2θ = 26° and 54°. These peaks are comparable to the peaks observed in graphite, which suggest that graphene still preserves the structure of carbon atoms and only the intensity and width of the peak in graphene have altered compared with graphite [29]. In the XRD pattern of the graft copolymer, a broad peak is observed, which indicates an irregular structure in the graft copolymer. The appearance of a broad peak associated with the copolymer and a sharp peak related to graphene in the nanocomposite suggests that the nanocomposite was successfully prepared. As well, the peak intensity associated with graphene is wider in the prepared nanocomposite pattern, this might be owing to the stacking of the graphene structure in the graft copolymer chains, which reduces crystallinity in the nanocomposite structure.

**Figure 4:**
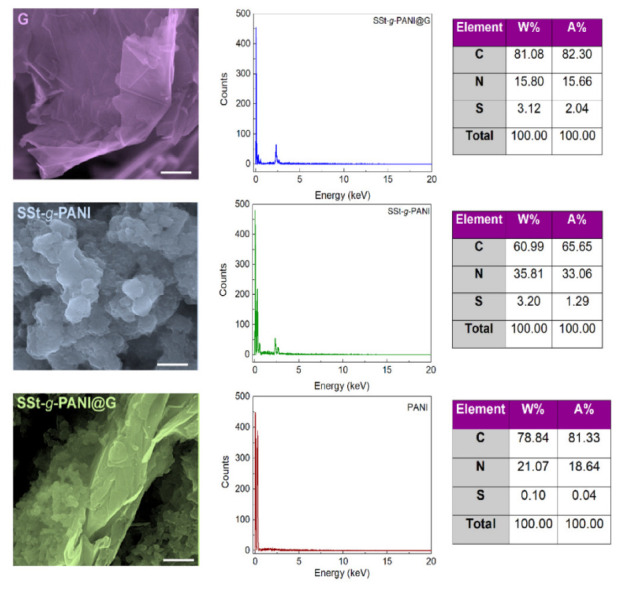
The FE-SEM images (scale 500 nm) of graphene (G), sulfonated starch-*graft*-polyaniline (SSt-*g*-PANI), and the sulfonated starch-*graft*-polyaniline copolymer@graphene (SSt-g-PANI@G) nanocomposite. EDX spectra and tabulated data of PANI, SSt-*g*-PANI, and the SSt-*g*-PANI@G nanocomposite.

The morphologies and particle size of G, SSt-*g*-PANI, and SSt-*g*-PANI@G nanocomposite were investigated by FE-SEM (Figure 4). The FE-SEM image of graphene illustrates its flaky structure, which has more wrinkles than graphite [25,30]. After linking polyaniline to SSt, the aggregated granular nanoparticles are observed. In the FE-SEM images of the nanocomposite sample, it can be seen that the nanoparticles are accumulated on the surface of the graphene flakes. EDX analysis was also conducted to determine the chemical composition of the nanocomposite (Figure 4). The results of this analysis displayed the presence of carbon, nitrogen, and a small number of sulfur elements attributed to the polyaniline and the ammonium persulfate structure, respectively which could be acceptable evidence of the PANI synthesis. In the EDX SSt-*g*-PANI copolymer, the increase in the percentage of sulfur reflects the attachment of the sulfone functional group to the starch. The elevation in the percentage of carbon along with other elements implies the effective synthesis of the nanocomposite. Since this analysis is relevant to a portion of the sample, the percentages derived from this analysis are different from the elemental analysis; hence, uniformity, non-uniformity, and other characteristics might be influential. The construction of the nanocomposite was further investigated via CHNSO analysis and the weight percentage of the elements was also determined. The results of elemental analysis for SSt, SSt-*g*-PANI copolymer, and SSt-g-PANI@G nanocomposite are presented in Table 1. The presence of the sulfur element in the SSt indicates its successful synthesis. The slight difference in the amount of sulfur is due to the presence of ammonium persulfate (as an initiator) in the copolymerization process. In addition, the presence of nitrogen in the copolymer sample is another reason for the successful synthesis of the graft copolymer. The increase in the carbon percentage in the nanocomposite also confirmed the proper dispersion and distribution of graphene nanoparticles in the structure of the nanocomposite. In general, the following results indicate the correct matching of the percentages of elements with the stoichiometry of the components used during polymerization and the success in preparing the nanocomposite.

The thermal stability of graphene (G), net polyaniline (PANI), sulfonated starch (SSt), sulfonated starch-*graft*-polyaniline (SSt-*g*-PANI), and the sulfonated starch-*graft*-polyaniline @graphene nanocomposite (SSt-*g*-PANI@G) was evaluated by TGA in the temperature range of 30 °C to 800 °C under an inert atmosphere (Figure S2). Due to the presence of hemiacetal rings, hydrogen intermolecular/intramolecular forces between polymer chains, and as a result of its dense and regular structure, net starch has high thermal resistance up to 350 °C [31]. According to the TGA curve of the SSt sample, a decrease in the thermal resistance of starch and two stages of weight loss in the temperature ranges of 150–200 °C and 250–400 °C can be seen. This weight loss occurs due to the reduction in intermolecular forces and the creation of a regular structure after the sulfonation process. In addition, the degradation temperature of net polyaniline illustrates decomposition in two steps. The first weight loss at 115 °C demonstrates the loss of absorbed moisture and the few oligomers present. The second weight loss occurs between 200 and 500 °C, indicating the polymer chain breakdown [32]. Further, by comparing the curves of SSt-*g*-PANI, SSt, and net polyaniline, an increase in the degradation temperature and thermal resistance of the SSt-*g*-PANI is observed. Net graphene has higher thermal stability among all samples and it is accompanied by only a little weight loss at a temperature of 110 °C [33,34]. Finally, when the degradation temperature and thermal stability of the SSt-*g*-PANI@G nanocomposite prepared are compared with other samples, a rise in the degradation temperature and thermal stability of the nanocomposite is observed. This stability is attributed to the existence of graphene nanoparticles as well as the strong link between nanoparticles with polyaniline chains and starch, which confirms the effective preparation of nanocomposite. 

### 3.2. Electrochemical Behaviors of SSt-g-PANI@G/Tyase/GCE

The electrochemical behavior of the bare electrode, modified electrode with nanocomposite, and immobilized Tyase on the modified electrode with nanocomposite were investigated by the cyclic voltammetry (CV) method for probing the feature surface-modified electrode and testing the kinetic barrier of the interface. Figure S3A illustrates the typical CVs of the bare electrode, modified electrode with nanocomposite, and modified electrode with nanocomposite and Tyase in 10 mL phosphate buffer 0.1 M (pH 6.8) at a scan rate of 100 mVs^−1^. The bare electrode shows no redox peak in its voltammogram, while the modified electrodes prepared by nanocomposite illustrate cathodic and anodic peaks. The anodic and cathodic peak currents gradually increase in the presence of the enzyme. The SSt-*g*-PANI@G/Tyase film on the activated GC electrode showed a pair of well-defined quasi-reversible peaks at about −200 and −100 mV vs Ag/AgCl at a scan rate of 100 mV s^−1^ (Figure S3A, green line). The formal potential (E°′) of SSt-*g*-PANI@G/Tyase/GCE was determined to be −150 mV vs. Ag/AgCl. This value is in the range of the E°′ values reported for Tyase from different sources and different methods of immobilization [35]. The cathodic current to anodic current ratio is close to one, indicating the stability and positive interaction of the nanocomposite with the electrode surface and the enzyme.

Polyaniline has the role of increasing conductivity, and graphene increases the sur-face-to-volume ratio and, as a result, more enzymes are loaded on the electrode surface. The negatively charged sulfonate groups in the starch, on the one hand, increase the solubility of the polymer, and on the other hand, suitable electrostatic interactions with the positive charges of amine groups on the surface of Tyase provide a suitable biocompatible microenvironment for enzyme stabilization. As a result, electron transfer between the tyrosinase enzyme and the electrode surface is improved, and the process is expedited; it also causes the enzyme to last longer on the surface of the electrode.

#### 3.2.1. pH Effect

**Figure 5:**
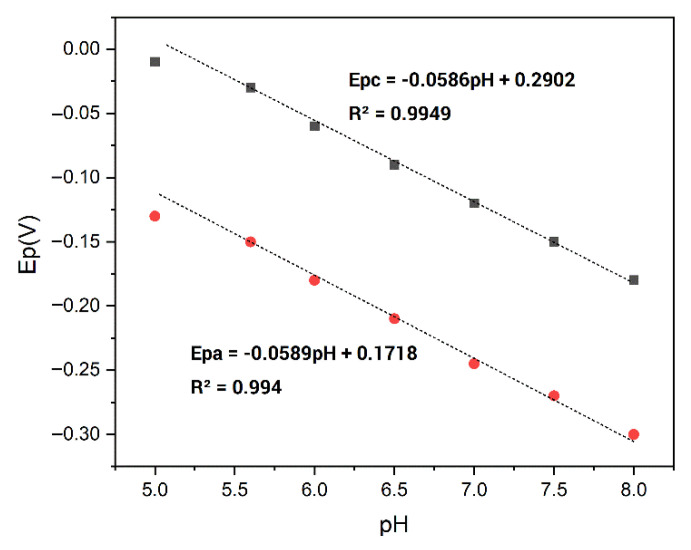
The linear relationship for the formal potentials of SSt-*g*-PANI@G/Tyase/GCE.

The pH influence on the SSt-*g*-PANI@G/ Tyase /GCE response was also investigated by cyclic voltammetry in phosphate buffers at a pH range of 5.6 to 8.0. The cathodic and anodic potentials (Epa and Epc) were shifted to the negative side when the pH increased (Figure S3B). This implies that proton transfer has a considerable impact on the redox process. Furthermore, the slopes of the formal cathodic and anodic potentials against pH values were −58.6 and −58.9 mV/pH, respectively. This slope is quite similar to the slope of the Nernst equation (−59 mV) for a two-proton coupled with a two-electron redox reaction process (Figure 5) [36]. This is probably due to the performance of protons in the reaction environment which affects the amino acids existing in the active site of the enzyme and causes them to be protonated or deprotonated.

#### 3.2.2. Scan Rate Effect

**Figure 6:**
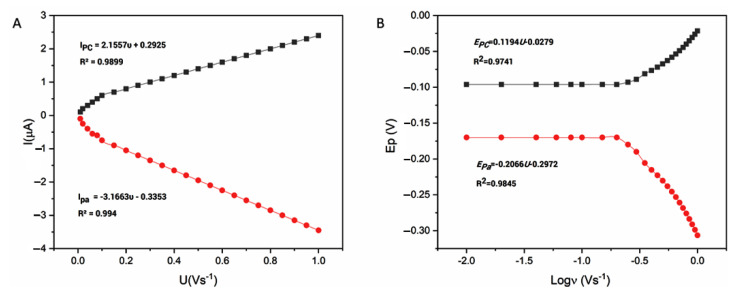
(**A**) The peak current against the scan rate of the SSt-g-PANI@G nanocomposite electrode in 10 mL phosphate buffer 0.1 M (pH 6.8) at different scan rates (10–1000 mV s^−1^); (**B**) the relationship of the anodic and cathodic peak potential against log ʋ.

To evaluate the kinetic parameters of the immobilized enzyme at SSt-*g*-PANI@G/ Tyase/GCE, the CVs of SSt-*g*-PANI@G /Tyase/GCE in 10 mL phosphate buffer 0.1 M (pH 6.8) at different scan rates from 10 to 1000 mV s^−1^ were examined (Figure S3C). When the scan rate increased, the redox peak currents (Ip) in the modified electrode with the nanocomposite were linearly increased. This can be due to the better stabilization of the nanocomposite on the electrode and its better overlap with the enzyme. The regression equations for anodic and cathodic peak currents were Ipa (µA) = −3.1663 ʋ (Vs^−1^)- 33.53(R^2^= 0.994) and Ipc (µA) = −2.1557 ʋ (Vs^−1^)+ 29.25 (R^2^= 0.9899), respectively (Figure 6A), indicating a surface-restricted electron transfer mechanism of SSt-*g*-PANI@G /Tyase /GCE which is expected for immobilized systems [37]. The amount of immobilized enzyme can be estimated according to the following Laviron equation [38]:

Ip = n2F2AΓʋ/4RT

where, Γ (mol cm^−2^) is the surface coverage of adsorbed Tyase, A (cm^2^) is the real electrode surface area, n is the number of electrons transferred in the rate-determining reaction, and the other symbols have their known concepts. Assuming a two-electron transfer and from the Ip vs. ʋ slope, the amount of protein molecules is estimated to be 3.38 × 10^−10^ mol cm^−2^. It is worth noting that this value is near to the reported theoretical one (1.89 × 10^−11^ mol cm^−2^) and indicated that approximately a thin layer of Tyase molecules takes the electrode reaction which is in agreement with the result of Log Ipc vs. Log ν.

The redox peak potentials illustrated a linear relationship with the logarithm of scan rate (log ʋ) ranging from 0.01 to 1 V s^−1^ at scan rates over 300 mVs^−1^ with the equations as Epc(V) = −0.1194 log ʋ (V s^−1^) − 0.0297 (R^2^= 0.9741) and Epa(V) = 0.2066 logʋ(V s^−1^)−0.2972 (R^2^ = 0.9845), respectively (Figure 6B), which revealed a quasi-reversible electrochemical system. The charge transfer coefficient (α) and electron transfer rate constant (ks) for immobilized Tyase onto SSt-*g*-PANI@G nanocomposite can be derived using Laviron’s equations by evaluating the variability of peak potential with scan rate [39]. The charge transfer coefficient (α) can be computed and found to be 0.45. The heterogeneous electron-transfer rate constant (ks) was calculated to be around 1.6 s^−1^. This number is almost two times quicker than the apparent electron transfer rate constant (0.9 s^−1^) determined in our previous work [40]. Considering the value of Ks, it can be concluded that the stabilization of the tyrosinase enzyme on the nanocomposite has increased the rate of electron transfer and made it faster. The possible reasons for the enhanced electron transfer may be ascribed to the good electrical conductivity of the nanocomposite and its microenvironment for an enzyme to undergo a facile electron transfer reaction [41,42].

#### 3.2.3. Catalytic Activity

**Figure 7:**
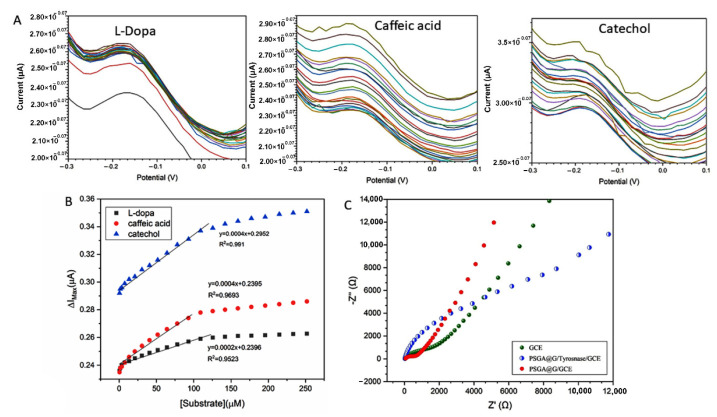
(**A**) Differential pulse voltammetry (DPV) of SSt-*g*-PANI@G/tyrosinase /GCE in the presence of L-dopa, catechol, and caffeic acid; (**B**) the Michaelis–Menten diagrams for the three substrates of L-dopa, caffeic acid, and catechol; (**C**) Nyquist plots of the bare GCE, SSt-*g*-PANI@G/GCE and SSt-*g*-PANI@G/Tyase/GCE in 1.0 mM [Fe(CN)_6_]^3−/4−^ redox probe and 0.1 M KCl as the supporting electrolyte. The frequency ranges of 0.1 to 100,000 Hz, with AC amplitude potentials of 0.005 V and DC amplitude of 0.3 V were applied.

Differential pulse voltammetry (DPV) was performed to investigate the activity of SSt-*g*-PANI@G/Tyase /GCE (Figure 7A). The peak currents of the as-prepared nanocomposite were examined in the presence of L-dopa, catechol, and caffeic acid. In this regard, the DPV response of the as-prepared SSt-*g*-PANI@G/Tyase/GCE at various concentrations of L-dopa was from 0.5 to 251.8 μM in 10 mL phosphate buffer 0.1 M (pH 6.8). By increasing the concentration of L-dopa substrate, the cathodic peak current increases, which indicates the suitable enzymatic activity of the SSt-*g*-PANI@G /Tyase/GCE. Due to the distance between the peak currents of the two initial concentrations, it is clear that this electrode responds better to low concentrations of L-dopa. Further, the calibration curve of L-dopa was linear at different concentrations (ranging from 0.5 to 109 µM) under optimal conditions. The regression equation is ipc = 0.0002 C L-dopa + 0.2396 (R^2^ = 0.9523). The low detection limit (LOD), relative standard deviation (RSD), and the apparent Michaelis–Menten constant (K_mapp_) of L-dopa were obtained at 15.0 µM, 0.001 µM, and 24.5 μM, respectively. 

The Lineweaver–Burk plots were drawn for different concentrations of catechol (51.7 µM, 64 µM, 78 µM, 93 µM, 109 µM, 125.5 µM, 142 µM, 159 µM, 177 µM, 195.8 µM, 214.8 µM, 233 µM, and 251.8 µM). Using this plot, the apparent Michaelis–Menten constant (K_mapp_) and the maximum measured current under saturated substrate conditions can be calculated [43]. The number of kinetic parameters such as apparent K_mapp_ and I_max_ was obtained at 3.22 μM and 0.27 μA, respectively. Similarly, the cathodic peak current of catechol increased with its concentration increasing from 0.5 to 251.8 μM in 10 mL phosphate buffer 0.1 M (pH 6.8). A linear regression equation was obtained as Ipc= 0.0004 C catechol + 0.2952 (R^2^ = 0.991) and the LOD and RSD were obtained at 11.25 and 0.0015 µM. Paying attention to the graph, the sensitivity of the biosensor is equal to its slope of 0.0004 µA/µM. The value of K_mapp_ here is much lower than those reported for the PANI–PPO film (146 μM) [27], the PPO cross-linked with PANI film (117 μM) [28], and the PANI/catechol biosensor (77.56 μM) [29] using catechol as the substrate, whereas it is very similar to the Tyase-SWCNTs/GCE (24.71 μM) [11].

The differential pulse voltammograms of caffeic acid were monitored at concentrations ranging from 0.5 to 251.8 µM in 10 mL phosphate buffer 0.1 M (pH 6.8). It is found that as the caffeic acid concentration enhances, the cathodic peak grows sharper in the system, indicating that the modified SSt-*g*-PANI@G/Tyase/GCE is enzymatically active. The linear range of 0.5 to 109 µM was derived from the differential pulse voltammogram in the equation of Ipc = 0.0004 C caffeic acid + 950.23 (R^2^= 0.9693). According to this diagram, the biosensor’s sensitivity is equal to its slope, which is equal to 0.0004 µA/µM. Finally, the LOD and RSD were calculated to be 13.5 and 0.0018 µM, respectively. The Lineweaver–Burk plots were also drawn at various caffeic acid concentrations (78 µM, 51 µM, 64 µM, 78 µM, 93 µM, 109 µM, 125.5 µM, 142 µM, 159 µM, 177 µM, 195.8 µM, 214.8 µM, 233 µM, and 251.8 µM). The apparent K_mapp_ and I_max_ were determined to be 6.25 µM and 0.3 µA, respectively. The linear range and LOD in this work are better than the direct immobilization of Tyase on GCE by Woodward’s reagent in our previous work [30].

By comparing the Michaelis–Menten diagrams for the three substrates of L-dopa, caffeic acid, and catechol, it can be seen that the higher slope and, as a result, the best response of the SSt-*g*-PANI@G/Tyase was obtained for catechol (Figure 7B).

The steps of the enzymatic reaction on the electrode surface are shown as follows [31]:

Catechol + Tyase (O_2_) → o-quinone + H_2_O (1)

o-Quinone + 2H^+^ + 2e^−^ → catechol (at electrode) (2)

**Figure 8:**
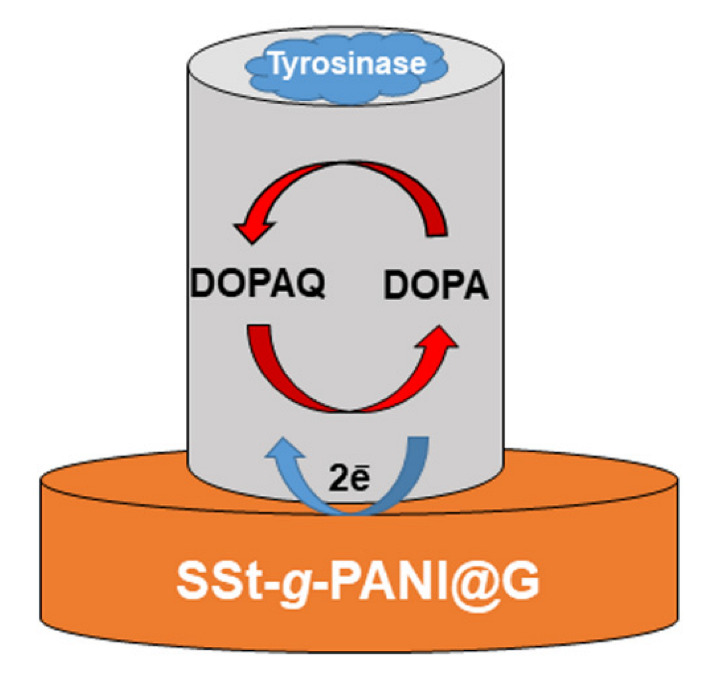
The principle of detecting L-dopa by using a SSt-*g*-PANI@G/Tyase/GCE.

Therefore, according to Figure 8, which shows the schematic of L-dopa oxidization to dopaquinone (DOPAQ), it can be seen that with SSt-*g*-PANI@G/Tyase /GCE-sensors, L-dopa can be easily electrocatalytic oxidized at the SSt-*g*-PANI@G/Tyase /GCE film to form DOPAQ which can be reduced at the electrode surface when a potential is applied to the electrode, after the exchange of two electrons (and two protons) to produce a Faradaic current [32]. In the case of biosensors, during the L-dopa oxidation steps, the oxidation states of the copper atoms of tyrosinase change to give different forms of the enzyme. Based on Solomon et al. [33], the resting state of the enzyme is mainly in the oxidized form [Tyase–Cu(II)] which can interact with diphenolic compounds such as L-dopa. The products of this catalytic reaction are the oxidized form of L-dopa (o-quinone) and the reduced form of the enzyme [Tyase –Cu(I)]. Therefore, by increasing the amounts of L-dopa, the concentration of [Tyase –Cu(I)] is also increased. This, in turn, causes a greater anodic peak current.

#### 3.2.4. Electrochemical Impedance Spectroscopy

Electrochemical impedance spectroscopy (EIS) is one of the most important electrochemical techniques for interrogating surface chemistries which can be applied to detecting interfacial binding events through impedance measurements (in ohms). EIS has advantages over other electrochemical methods, such as that it is a steady-state technique, uses small signal analysis, and that it can investigate signal relaxation over a very wide range of applied frequencies with commercial usage [44,45]. On the other hand, EIS can be applied for assessing the electrical characteristics of nanomaterials, detecting and investigating the behavior of the electrode surface by delivering a sinusoidal voltage across a wide frequency range, and measuring the current with the least negative impact [46]. Figure 7C illustrates the Nyquist plots for the bare GCE, SSt-*g*-PANI@G/GCE, and SSt-*g*-PANI@G/Tyase/GCE in the presence of 1 mM [Fe(CN)_6_]^3−/4−^ redox and 10 mL phosphate buffer 0.1 M at pH 6.8. As can be seen, the size of the Nyquist semicircle of the bare electrode is bigger than that of the electrode with the as-prepared nanocomposite, indicating that it has a higher charge-transfer resistance than the electrode with the nanocomposite [47]. As a result of the nanocomposite’s stability, the electron transfer resistance (Ret) decreased, owing to the nanocomposite’s favorable conductivity. Therefore, the charge transfer process between the electrolyte and the electrode surface was expedited. The impedance spectrum of a modified electrode (SSt-*g*-PANI@G /Tyase) shows a considerable increase in the diameter of the semicircle, indicating that the tyrosinase enzyme is correctly established on the electrode surface and preventing electron transport. Overall, the results indicate that the produced nanocomposite is a plausible option for developing biosensors.

#### 3.2.5. The Temporal Stability of Sensor Performance

The stability influence of the SSt-*g*-PANI@G/Tyase/GCE was explored using cyclic voltammetry in 10 mL phosphate buffer 0.1 M at pH 6.8 for 17 days. Figure S3D reveals that there is no substantial alternation in the current response of this biosensor after the period. More precisely, the stability analysis of tyrosinase enzyme activity reveals that after 17 days, the modified electrode retained 83.07% of its tyrosinase enzyme activity, indicating the time stability of this sensor.

## 4. Conclusions

In this work, we showed the feasibility of developing a biosensor for total catechol, L-dopa, and caffeic acid detection by using novel electrochemical nanoplatforms based on the immobilization of tyrosinase on a sulfonated starch-*graft*-polyaniline@graphene nanocomposite (SSt-***g***-PANI@G). Several analyses such as FTIR, XRD, EDX, and CHNSO approved the preparation of the nanocomposite. The SSt-***g***-PANI@G was prepared using an in situ copolymerization process and the tyrosinase enzyme was subsequently immobilized on its surface through the casting approach. The SSt-g-PANI@G nanocomposite was provided a friendly environment for tyrosinase immobilization, thus enhancing the catalytic activity of the enzyme and, at the same time, showing an improved platform conduction pathway owing to the higher conductivity compared with a pristine electrode. The SSt-***g***-PANI@G nanocomposite matrix showed superior electrochemical performances, which can be attributed to intermolecular interaction (hydrogen bonding) between sulfuric acid and hydroxyl groups in SSt, amine groups in PANI, and also a high surface-to-volume ratio of graphene. It was discovered that the apparent K_mapp_ of L-dopa is lower than that of the other two substrates because L-dopa is the main substrate of the tyrosinase enzyme; therefore, the tyrosinase has a greater affinity for it and the sensitivity of the SSt-g-PANI@G/Tyase/GCE biosensor to the catechol substrate is higher than other ones. In addition, due to the simple structure of catechol, the catalytic activity of the enzyme was better than L-dopa and caffeic acid. Thus, we conclude that the SSt-g-PANI@G nanocomposite is a good candidate for the construction of a tyrosinase biosensor.

## Figures and Tables

**Table 1:** The result of elemental analysis (CHNSO) of sulfonated starch (SSt), sulfonated starch- *graft*-polyaniline (SSt-*g*-PANI), and the sulfonated starch-*graft*-polyaniline@graphene (SSt-*g*-PANI@G) nanocomposite.

Sample	%C	%O	%H	%N	%S
SSt	58.71	31.04	8.14	-	2.11
SSt-*g*-PANI	46.40	38.60	6.04	5.78	3.18
SSt-*g*-PANI@G	60.82	21.94	6.46	7.68	3.10

## Data Availability

Data available on request.

## Supplementary Materials

The following supporting information can be downloaded at: www.mdpi.com/article/10.3390/bios12110939/s1, Figure S1: FTIR spectrum of polyaniline (PANI); Figure S2: The thermal stability of graphene (G), net polyaniline (PANI), sulfonated starch (SSt), sulfonated starch-*graft*- polyaniline (SSt-*g*-PANI), and the sulfonated starch-*graft*-polyaniline@graphene nanocomposite (SSt-*g*-PANI@G); Figure S3: (A) CVs of different electrodes in 10 mL phosphate buffer 0.1 M (pH 6.8) at a scan rate of 100 mVs^−1^ for SSt-g-PANI@G nanocomposites. Bare electrodes (blue curves), modified electrodes with nanocomposite (orange curves), and modified electrodes with nanocomposite and tyrosinase enzyme (green curves). (B) The CVs of the effect of different pHs (6.5 to 8) on the electrochemical behavior of modified SSt-*g*-PANI@G/Tyase/GCE electrode at a scanning speed of 100 mV/S^−1^. Bare electrode (black), second day (red), fourteenth day (blue), and seventeenth day (green) of SSt-*g*-PANI@G/Tyase /GCE electrode. (C) Cyclic voltammetry of the different electrodes in 10 mL phosphate buffer 0.1 M (pH 6.8) at different scan rates (10–1000 mV s^−1^). (D) Time stability diagram of SSt-*g*-PANI@G/ Tyase /GCE biosensor obtained from in situ (circle) and solution mixing (square) methods during 17 days.
