# Supplementary material for: Sulfonated Starch-Graft-Polyaniline@Graphene Electrically Conductive Nanocomposite: Application for Tyrosinase Immobilization
Source: Biosensors (Basel). 2022 Oct 28;12(11):939. doi: 10.3390/bios12110939 (PMC9688083; doi:10.3390/bios12110939)
Supplement: Supplementary file 1 [file biosensors-12-00939-s001.zip › biosensors-1959811-supplementary.pdf]

biosensors

Sulfonated Starch-*Graft*-Polyaniline@Graphene Electrically Conductive Nanocomposite: Application for Tyrosinase Immobilization

[1] School of Chemistry, Damghan University, Damghan 36716-41167, Iran
[2] School of Biology, Damghan University, Damghan 36716-41167, Iran
[3] Centre for Materials Interface, Istituto Italiano di Tecnologia, Viale Rinaldo Piaggio 34, Pontedera, 56025 Pisa, Italy
* Correspondence: e.nazarzadeh@du.ac.ir (E.N.Z.); pooyan.makvandi@iit.it (P.M.)

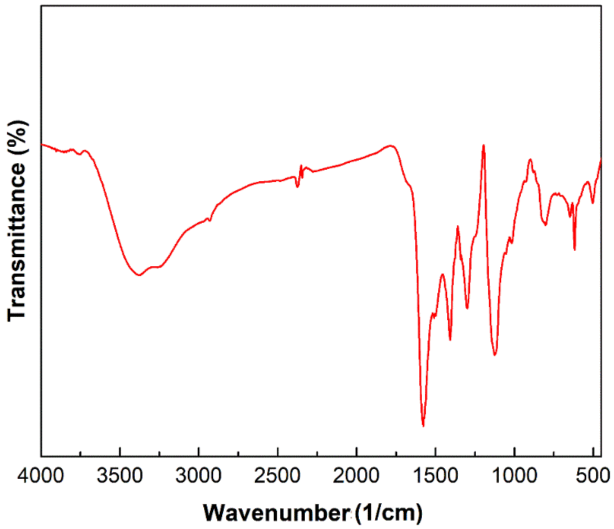

Figure S1. FTIR spectrum of polyaniline (PANI).

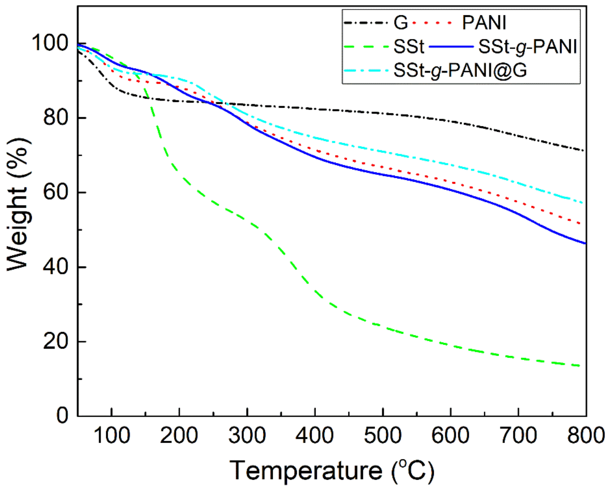

Figure S2. TGA themograms of graphene (G), net polyaniline (PANI), sulfonated starch (SSt), sulfonated starch graft polyaniline copolymer (SSt-g-PANI) and sulfonated starch graft polyaniline copolymer@graphene nanocomposites (SSt-g-PANI@G).

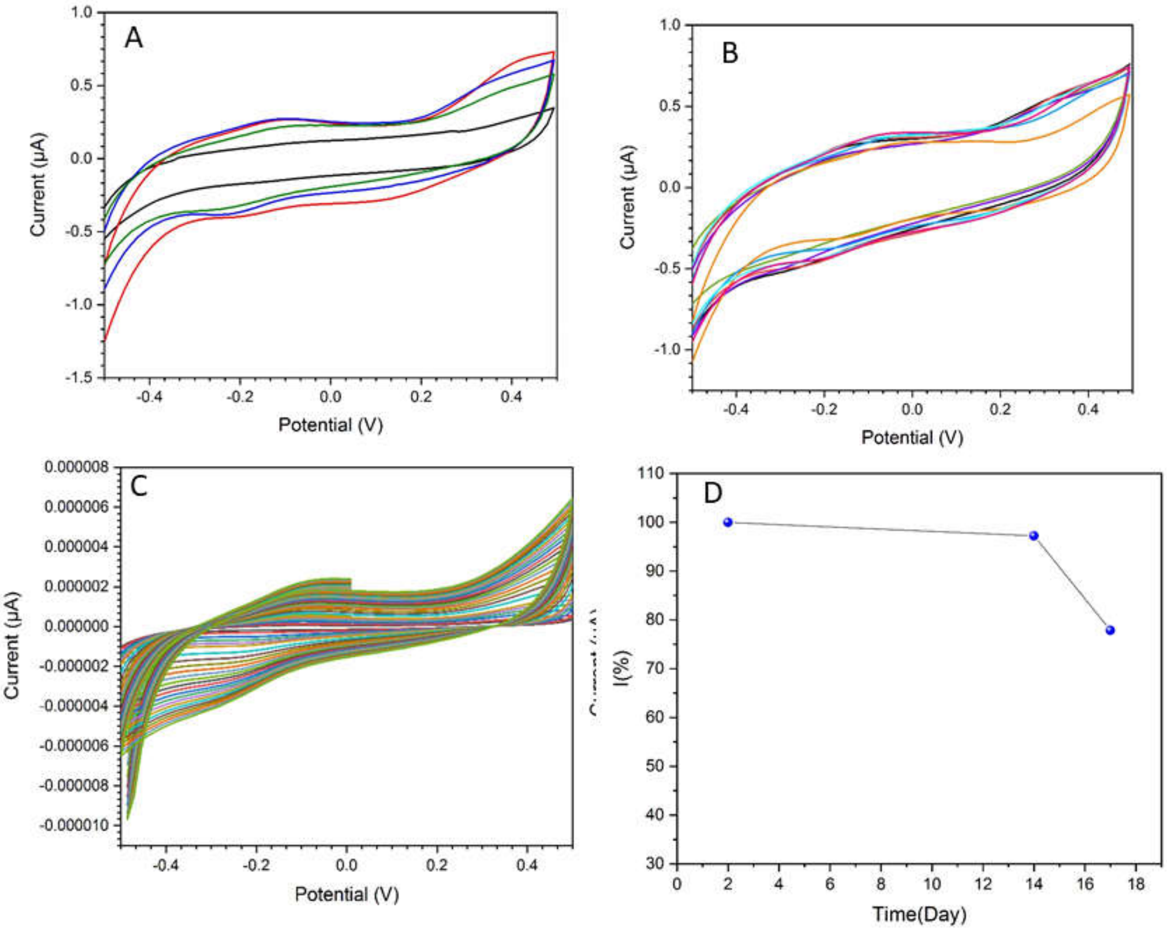

Figure S3. (**A**) CVs of different electrodes in 10 mL phosphate buffer 0.1 M (pH 6.8) at a scan rate of 100 mVs^{-1} for SSt-g-PANI@G nanocomposites. Bare electrodes (blue curves), modified electrodes with nanocomposite (orange curves), and modified electrodes with nanocomposite and tyrosinase enzyme (green curves)**.** (**B**) The CVs of the effect of different pHs (6.5 to 8) on the electrochemical behavior of modified SSt-g-PANI@G/tyrosinase/GCE electrode at a scanning speed of 100mV/S^{-1}. Bare electrode (black), second day (red), fourteenth day (blue), and seventeenth day (green) of SSt-g-PANI@G/tyrosinase/GCE electrode. **(C)** Cyclic voltammetry of the different electrodes in 10 mL phosphate buffer 0.1 M (pH 6.8) at different scan rates (10-1000 mV s^{-1}). (**D**) Time stability diagram of SSt-g-PANI@G /tyrosinase/GCE biosensor obtained from *in-situ* (circle) and solution mixing (square) methods during 17 days.
